# Healthcare professionals’ views on training, standards, and resources for extracorporeal membrane oxygenation: a letter to the editor

**DOI:** 10.3325/cmj.2026.67.132

**Published:** 2026-04

**Authors:** Huseyin Durmaz, Semiha Durmaz

**Affiliations:** 1 Department of Cardiovascular Surgery, Konya City Hospital, Konya, Turkey; durmazztoprak@gmail.com; 2 Department of Internal Medicine, Konya City Hospital, Konya, Turkey

To the Editor,

We have read with interest the article entitled “Opinions of healthcare professionals on training, standards, and resources for extracorporeal membrane oxygenation: a cross-sectional study,” recently published in the *Croatian Medical Journal* by Permenov et al (1). The study offers a current and important contribution to addressing the issues of training, standards, resource adequacy, and accessibility related to the use of extracorporeal membrane oxygenation (ECMO).

This valuable research shows that a significant portion of health care professionals accept the clinical services of ECMO, but nevertheless, challenges remain regarding the accessibility of ECMO, the breadth of training, and institutional preparedness. Indeed, safe and sustainable application of ECMO depends not only on technological advancements but also on standardized training modules, disaggregated assessment, and the formation of appropriate teams (2). The study adds to the literature by highlighting practical obstacles to ECMO implementation such as cost, equipment support, and labor shortages. However, the following study limitations warrant cautious interpretations of the findings. First, due to its cross-sectional survey design, the study is based on self-reports rather than on objective indicators of ECMO capacity or institutional performance. Therefore, the use of more cautious language would be appropriate when referring to perceived problem areas rather than causal consequences of existing limitations, particularly in the Discussion section.

Furthermore, a single-setting design limits the generalizability of the findings. Differences in health care system characteristics, referral systems, reimbursement policies, and access to trained personnel across countries significantly affect the regulations and practices related to ECMO. Therefore, a broader interpretation of the results, highlighting the differences more clearly, could enhance the interpretive power.

Finally, while the study successfully reveals shortcomings in ECMO training and certifications, a clearer separation between individual knowledge deficiencies and institutional, national, or regional system capacity differences could further strengthen the practical and policy implications of its usefulness.
